# Notes and News

**Published:** 1901-03-23

**Authors:** 


### Notes and News.

### HOSPITAL MEETINGS.

National Hospital for Paralysed and Epileptic.—Special meeting, Saturday, March 23rd, at 12 noon.

Royal Sea Bathing Infirmary, Margate.—Annual meeting, Monday, March 25th, at 3 p.m. at 30 Charing Cross.

West London Hospital, Hammersmith.—Annual meeting, Monday, March 25th, at 5 p.m.

Poplar Hospital for Accidents.—Annual meeting, at the Hospital, March 26th, at 5.20 p.m.

St. Peter's Hospital, Henrietta Street, W.C.—Annual meeting, Tuesday, March 26th, at 5 p.m.

Royal Eye Hospital, St. George's Circus.—Annual meeting, Wednesday, March 27th, at 4 p.m.

British Lying-In Hospital, Endell Street, W.C.—Annual meeting, Thursday, March 28th, at 5 p.m.

The King has received an address of condolence and congratulation from the Medical Graduates' Association of Brussels.

Sir William MacCormac who was presented with the honorary freedom and livery of the Leathersellers' Company, and in whose honour a banquet was held on March 13th, will also receive the freedom of the Barbers' Company on March 26th.

In view of the exhibition so shortly to be opened in Glasgow, strenuous efforts are being made to arrest the smallpox epidemic, more especially by vaccination. A large number of students are being employed to go from house to house in the poorer districts to re-vaccinate the inhabitants. Primary vaccination can only be effected by registered medical men, and a large number of these are also being invited to join the crusade.

Sir James Willcocks in his latest dispatch describing the operations of the troops in Ashanti, pays a high compliment to the principal medical officer, Dr. McDowell. He states that Dr. McDowell was given an absolutely free hand, the supplies asked for were sent promptly by the Home Government, and the arrangements were as good as they could be in the most difficult circumstances. He also commends Dr. Murray of the Gold Coast Medical Service, and Dr. Gray of the British Central Africa Medical Service.

The second reading of the Bill for the exemption of hospitals from rates has been postponed until April 24th.

The committee charged with the arrangements of the Naval and Military Exhibition which will be held this summer at the Crystal Palace, have presented 50,000 guinea season tickets to the various societies which relieve naval and military men and their families.

Dr. Charles Wilberforce Daniels has been appointed as superintendent and medical tutor at the London School of Tropical Medicine in succession to Dr. Rees. Dr. Daniels has gained much experience in British Guiana where he was attached to the Colonial Medical Service.

The medical board appointed to inquire into the condition of the *Britannia* reports that the admissions on the sick list from diseases which may depend upon the sanitary condition of the ships have shown a steady and marked diminution during the past five years, while there has not been a single case of enteric fever, nor has any wound become septic during that period. They are further quite satisfied that the recent outbreak of influenza among the cadets was introduced from without and had nothing whatever to do with the sanitary condition of the ships.

In the Blue Book on the health of the Navy for 1899 which has just been issued Surgeon J. G. Fowler gives some interesting medical facts concerning the doings of the Naval Brigade from the *Powerful* during the siege of Ladysmith. He describes the great difficulty which was experienced in filtering the river water from the amount of mud which it contained, and goes on to say that ultimately the engineer of the Naval Brigade improvised a distilling apparatus. This officer on December 11th commenced to erect distillers in the railway station yard, employing the unused locomotives for the purpose, and thus by degrees all the troops were supplied with good drinking water, although its goodness was doubtless somewhat modified by the lack of water-carts, which made it necessary to use the same carts for the drinking water as were employed for the washing water, which was a pity.

At the meeting of the St. Saviour's Guardians, held last week, a very severe letter from the Local Government Board was read in regard to the disclosures recently made as to the acceptance of money by relieving officers from medical men and proprietors of licensed houses in connection with the certifying of lunatics and their admission into asylums. So seriously did the Board regard the matter that they had felt much doubt whether any officer concerned could be permitted to remain in office. "Under all the circumstances, however, they have decided, though with much hesitation, not to insist on the officers ceasing to hold office," but they give them a most serious warning, and it is to be hoped that such a scandal will not arise again. Everyone will agree with the Board that although the practice of receiving money from asylum proprietors may not have caused any persons which were not insane to have been sent to any of the asylums in question, "the whole tendency of the system is dangerous, and it is essential that it should cease."

